# Long-term growth and final adult height outcome in childhood-onset systemic lupus erythematosus

**DOI:** 10.1186/s12969-022-00663-0

**Published:** 2022-01-24

**Authors:** Lalita Ponin, Preamrudee Poomthavorn, Kwanchai Pirojsakul, Butsabong Lerkvaleekul, Sirisucha Soponkanaporn, Niyata Chitrapazt, Soamarat Vilaiyuk

**Affiliations:** 1 Division of Rheumatology, Department of Pediatrics, Faculty of Medicine Ramathibodi Hospital, Mahidol University, 270 Rama VI Road, Ratchathewi, Bangkok, 10400 Thailand; 2 Department of Radiology, Faculty of Medicine Ramathibodi Hospital, Mahidol University, Bangkok, Thailand

**Keywords:** Corticosteroids, Connective tissue disease, Pediatric, Growth impairment, Children, Puberty

## Abstract

**Background:**

Growth impairment is the most common complication in patients with childhood-onset systemic lupus erythematosus (cSLE). There are limited data on risk factors affecting growth development in Asian patients with cSLE. This study aimed to determine the predictors of growth impairment in such patients.

**Methods:**

All SLE patients aged < 15 years diagnosed in Ramathibodi Hospital between 2006 and 2016 were enrolled in a retrospective cohort study. Baseline characteristics, including height, weight, clinical manifestations, disease activity score, and medications, were reviewed from medical records from the time at diagnosis to achievement of final adult height (FAH). Age at menarche in girls, adult voice appearance in boys, and parental height were collected by interview. Parent-adjusted FAH (PaFAH) *Z*-score was calculated as the difference between FAH *Z*-score for chronological age of the patients and their mid parental height-*Z* score. The patients were classified into two groups: (1) normal growth (PaFAH *Z*-score ≥ − 1.5, 2) growth impairment (PaFAH *Z*-score < − 1.5). Descriptive statistics and logistic regression analysis were used to analyze the data.

**Results:**

Of 106 cSLE patients, 19 (18%) were male and 87 (82%) were female. The mean age at study enrollment was 20.6 ± 3.0 years, mean age at diagnosis 12.1 ± 2.3 years, and mean age at achievement of FAH 17.5 ± 1.9 years. Growth impairment was found in 23.6% of patients (52.6% in boys and 17.2% in girls). Predictors of growth impairment were male sex, duration of disease before menarche in girls and adult voice appearance in boys, and cumulative corticosteroid dose (prednisolone equivalent) ≥230 mg/kg received before the late phase of puberty, with odds ratios of 7.07 (95%CI 2.11–23.74), 1.26 (95% CI 1.02–1.56), and 6.99 (95%CI 1.63–30.02), respectively.

**Conclusions:**

One-fourth of cSLE patients developed growth impairment, which mostly affected male patients. Longer duration of disease before the late phase of puberty and corticosteroid dose ≥230 mg/kg received before the late phase of puberty were factors predictive of growth impairment.

## Background

Childhood-onset systemic lupus erythematosus (cSLE) is a common connective tissue disease involving several organ systems and accounts for 10–20% of all cases of systemic lupus erythematosus (SLE) [1]. More severe than adult-onset SLE, cSLE leads to a higher mortality rate and needs more intensive treatment. Given that the survival rate in patients with SLE has improved significantly during the past few decades from 42 to 72% to 95% [2], quality of life is a growing concern among the increasing number of survivors.

One of the most common adverse outcomes of cSLE and its treatment is growth impairment, which is potentially irreversible and can affect patients’ quality of life. Factors that contribute to growth impairment in these patients include prolonged duration of the disease, disease severity, age at disease onset, suboptimal nutrition, and use of medications, especially corticosteroids [3], which constitute the mainstay of SLE therapy. The most well-known adverse effect of corticosteroid use during childhood is growth suppression, which is secondary to their direct effect on the growth plate and the reduction of chondrocyte proliferation. Additionally, corticosteroids may cause growth delay through modification of gonadal function, leading to delayed puberty [4]. The higher the dose and the longer the course of corticosteroid use, the more severe are the expected adverse effects. A previous study in Caucasians with cSLE reported that age at first visit of 13.4 years or younger and cumulative corticosteroid dose of more than 426 mg/kg were risk factors for failure to grow [3]. A Canadian study showed that the height *Z*-score of patients receiving moderate- to high-dose corticosteroids decreased and did not recover within 18 months of therapy [5]. Other studies demonstrated that female sex, age at diagnosis of 11–13 years, and having pre-existing growth failure at the time of diagnosis were factors associated with growth failure following treatment [6, 7]. Ethnicity was an additional factor that influenced the achievement of final adult height (FAH) in cSLE patients in a multi-ethnic study [8]. Asian patients usually have more severe disease and thus receive more aggressive treatment [9]. However, data on growth trajectory in Asians during treatment while growing to FAH and their association with the cumulative dose of corticosteroids, which has been reported to affect growth, remain limited. Against this background, we performed this study to determine the FAH outcome and the predictors that affect growth trajectory and FAH in cSLE patients.

## Methods

This retrospective cohort study enrolled SLE patients who were diagnosed before the age of 15 years and were regularly treated at the Faculty of Medicine, Ramathibodi Hospital between 2006 and 2016. All patients met the diagnostic criteria of SLE, according to either the 1997 American College of Rheumatology classifications criteria for SLE [10] or the Systemic Lupus International Collaborating Clinics 2012 classification criteria for SLE [11]. This study excluded patients who had known vertebral compression fracture and patients who were lost to follow-up or transferred to other hospitals. The study was approved by the Ethics Committee of the Faculty of Medicine, Ramathibodi Hospital, Mahidol University, and written informed consent was obtained from patients prior to their enrollment.

### Data collection

Demographic data, including age, sex, height, weight, body mass index (BMI), disease duration, clinical manifestations, medications, laboratory data including complete blood count, urine protein-to-creatinine ratio, erythrocyte sedimentation rate, and complement levels, and presence of autoantibodies including anti-nuclear antibody and anti-double-stranded DNA (anti-dsDNA), were collected. Owing to the retrospective nature of this study, assessment of pubertal status during the follow-up period was lacking. Therefore, age at menarche in girls and age at first appearance of adult voice in boys were collected by interview and used as an indicator of the late phase of puberty [12]. Mid-parental height (MPH) was determined to estimate genetic height potential. The parent’s height was collected by interview or measured in the clinic. MPH is calculated in males by adding 6.5 cm to the mean of parental height ([mother’s height + father’s height]/2 + 6.5 cm) and in females by subtracting 6.5 cm from the mean of parental height ([mother’s height + father’s height]/2–6.5 cm).

With regard to height outcome, height, weight, and the parameters of disease activity were collected every 6 months for the first 2 years of follow-up. Subsequently, these data were collected annually until FAH was achieved. FAH was defined as having either height velocity of less than 1 cm per year for at least 1 year or bone age of 15 years or greater in girls and 17 years or greater in boys. Assessment of bone age was determined by a specialist in musculoskeletal radiology using the Greulich and Pyle method [13].

The Modified Systemic Lupus Erythematosus Disease Activity Index 2000 (Modified SLEDAI-2 K) [14] was used for assessment of disease activity. Using the trapezoidal rule, cumulative Modified SLEDAI-2 K scores over time were determined by summing the scores serially obtained between the date at diagnosis and the date of achieving FAH [15].

Disease duration was defined as the period from the date at diagnosis to the date of achieving FAH. Regarding treatment, data on the medications, including hydroxychloroquine, corticosteroids, mycophenolate mofetil, cyclophosphamide, azathioprine, calcium carbonate, and vitamin D, were collected. The cumulative doses of corticosteroids (prednisolone equivalent) and cyclophosphamide were calculated by the sum of the medication doses at each visit.

### Anthropometric data

Height (cm) and weight (kg) were collected at each clinic visit. Height was measured in the morning by trained nurses using a wall-mounted stadiometer. BMI was calculated as weight (kg) divided by height (m) squared and expressed as *Z*-score based on World Health Organization growth references [16]. Standardization of height and weight were adjusted according to chronological age (years) and expressed as *Z*-scores based on the data of the Ministry of Public Health, Thailand [17]. Parent-adjusted FAH (PaFAH) *Z*-score was calculated as the difference between FAH *Z*-score for chronological age of the patients and their MPH-*Z* score [3]. The patients were then classified into two groups: (1) normal growth group (PaFAH *Z*-score of − 1.5 or greater) and (2) growth impairment group (PaFAH *Z*-score of less than − 1.5) [18].

### Statistical analysis

Descriptive data were reported for normally distributed variables as mean and standard deviation (SD), for non-normally distributed variables as median and interquartile range (IQR), and frequency as a percentage. Parameters of cSLE between normal growth and growth impairment groups were compared using independent-sample *t*-test and Mann–Whitney U test for continuous data, and chi-squared test and Fisher’s exact test for categorical data. The area under the receiver-operating characteristic curve was calculated to determine the cutoff value of cumulative doses of corticosteroids in predicting growth impairment. Logistic regression analysis was performed to determine the predictors of growth impairment in cSLE patients and presented as odds ratio (OR). Significance was set at *P* < 0.05. Statistical analysis was performed using SPSS statistical software version 21 (IBM, Armonk, NY, USA).

## Results

Out of a total of 140 patients with cSLE who reached FAH, 34 patients were excluded (1 died, 6 had vertebral compression fractures, and 27 were transferred to other hospitals). Therefore, 106 Thai patients with cSLE were enrolled in this study, of whom 39 (37%) had bone age assessment. Nineteen (18%) patients were male and 87 (82%) were female. The age (mean ± SD) at study enrollment was 20.6 ± 3.0 years and mean age at diagnosis of cSLE was 12.1 ± 2.3 years. The mean age at achievement of FAH was 17.5 ± 1.9 years and mean disease duration 5.5 ± 2.7 years. Most of the cSLE patients (64.2% of the total 106) had renal involvement (54.4%of whom had lupus nephritis class III, IV, or V), followed by musculoskeletal (51.9%), hematological (44.3%), and neurological (30.2%) involvement. All patients received corticosteroid treatment accompanied by vitamin D and calcium carbonate supplementation. Around 45% additionally received cyclophosphamide, and 55% received azathioprine. Twenty percent of these patients received mycophenolate mofetil and 15% received methotrexate, while a few (4%) received cyclosporine. Baseline disease activity was at a high level according to the average Modified SLEDAI-2 K score.

### Growth outcomes

There were 25 (23.6%) patients (10 boys and 15 girls) in the growth impairment group. Median (IQR) FAH of all cSLE patients was 1.6 (− 7.1, 0.9) cm lower than MPH, with a PaFAH *Z*-score of − 0.3 (− 1.5, 0.2). Age (mean ± SD) at FAH attainment of patients in the growth impairment group (17.7 ± 1.5 years) and the normal growth group (17.5 ± 1.9 years) were similar. Median (IQR) PaFAH *Z*-score of patients in the growth impairment and normal growth groups were − 2.3 (− 2.7, − 1.9) and 0.0 (− 0.5, 0.4), respectively. The baseline characteristics, clinical manifestations at diagnosis, and medication use between patients in both groups were not significantly different except for a higher male/female ratio, lower height *Z*-score at diagnosis, and lower weight *Z*-score at achievement of FAH, in the growth impairment group in comparison with those in the normal growth group (Table 1). Patients in the growth impairment group experienced a longer time from onset of disease to the late phase of puberty in comparison with the normal growth group (median [IQR] disease duration, 2.0 [1.4, 4.0] years vs. 1.0 [0, 3.0] years, respectively; *P* = 0.034).

**Table 1:** Clinical characteristics of cSLE patients with normal growth and growth impairment (*N* = 106)

	All patients	Normal growth	Growth impairment	***P*** value
	***N*** = 106	***n*** = 81	***n*** = 25	
**Patient characteristics**				
Male/Female, *n* (%)	19/87 (18/82)	9/72 (11/89)	10/15 (40/60)	0.002^a^
Age at diagnosis (y)	12.1 ± 2.3	12.2 ± 2.4	11.4 ± 2.1	0.122
Age at FAH (y)	17.5 ± 1.9	17.5 ± 1.9	17.7 ± 1.5	0.553
Age at menarche/adult voice appearance (y)	13.7 ± 1.6	13.6 ± 1.4	14.2 ± 1.9	0.084
Disease duration (y)^c^	5.5 ± 2.7	5.2 ± 2.7	6.3 ± 2.5	0.063
Disease duration before menarche/adult voice appearance (y)^b^	1.7 (0, 3.1)	1.0 (0.0, 3.0)	2.0 (1.4, 4.0)	0.034^a^
Cumulative Modified SLEDAI-2 K scores^b^	138.0 (72.0, 260.3)	168.0 (73.5, 262.5)	120.0 (69.0, 286.5)	0.766
**Clinical manifestations at diagnosis**				
Modified SLEDAI-2 K score	16.0 ± 10.6	15.5 ± 10.7	17.6 ± 10.6	0.721
Neurological, *n* (%)	32 (30.2)	24 (29.6)	8 (32.0)	0.821
All renal involvement, *n* (%)	68 (64.2)	51 (63.0)	17 (68.0)	0.646
Lupus nephritis class III, IV, V, *n* (%)	37 (34.9)	29 (35.8)	8 (32.0)	0.727
Musculoskeletal, *n* (%)	55 (51.9)	42 (51.9)	13 (52.0)	0.990
Hematological, *n* (%)	47 (44.3)	37 (45.7)	10 (40.0)	0.617
**Anthropometric data at diagnosis and at FAH attainment**				
Weight at diagnosis (*Z*-score)^b^	− 0.1 (− 0.8, 1.0)	0.2 (− 0.8, 1.4)	− 0.5 (− 1.0, 0.6)	0.125
Weight at FAH (*Z*-score)^b^	1.5 (−0.7, 3.0)	1.3 (−0.5, 3.6)	− 0.4 (− 1.4, 1.8)	0.015^a^
Height at diagnosis (*Z*-score)^b^	−0.3 (−1.0, 0.7)	−0.1 (− 0.8, 0.7)	− 0.6 (− 1.8, 0.2)	0.029^a^
Final height (*Z*-score)^b^	−0.1 (−1.3, 0.4)	0.04 (− 0.8, 0.7)	− 1.7 (−2.7, − 0.9)	< 0.001^a^
BMI at diagnosis, (kg/m^2^) (*Z*-score)^b^	−0.4 (−1.3, 1.0)	−0.2 (−1.3, 1.0)	2.0 (−1.3, 0.9)	0.204
BMI at FAH (kg/m^2^) (*Z*-score)^b^	−0.3 (− 0.7, 1.5)	0.5 (− 0.8, 1.6)	0.09 (− 0.6, 1.0)	0.634
Parent adjusted FAH (*Z*-score)^b^	−0.3 (−1.5, 0.2)	0.0 (−0.5, 0.4)	−2.3 (−2.7, −1.9)	< 0.001^a^
Difference between FAH and MPH (cm)^b^	−1.6 (−7.1, 0.9)	0.0 (−2.7, 2.1)	−11.7 (− 13.3, −9.5)	< 0.001^a^
**Laboratory data at diagnosis**				
Hematocrit (%)	30.0 ± 6.9	29.7 ± 7.2	30.8 ± 6.0	0.559
White blood cell count (× 10^3^ cells/mm^3^)^b^	4.7 (3.3, 6.9)	4.7 (3.3, 6.5)	4.8 (3.4, 8.1)	0.809
Absolute lymphocyte count (× 10^3^ cells/mm^3^)^b^	1.4 (1.0, 2.2)	1.3 (1.1, 2.1)	1.6 (0.9, 2.2)	0.710
Platelet count (×10^3^ cells/mm^3^)^b^	215.0 (114.0, 297.3)	212.0 (103.8, 293.0)	241.0 (122.5, 305.5)	0.554
Erythrocyte sedimentation rate (mm/h)^b^	56.0 (26.0, 83.3)	57.0 (25.0, 85.5)	53.0 (27.0, 79.5)	0.498
Anti-dsDNA (IU/mL)^b,d^	234.2 (97.4, 562.5)	271.9 (101.6, 577.2)	229.3 (70.2, 345.9)	0.510
C3 levels (0.9–1.8 g/L)^b^	0.6 (0.4, 1.0)	0.5 (0.3, 1.0)	0.8 (0.4, 1.0)	0.291
C4 levels (0.1–0.4 g/L)^b^	0.07 (0.05, 0.2)	0.07 (0.04, 0.2)	0.12 (0.06, 0.2)	0.092
25-OH vitamin D levels (ng/mL)^e^	33.78 ± 8.34	33.42 ± 7.14	34.93 ± 11.44	0.441
**Medications**				
Prednisolone, *n* (%)	106 (100%)	81 (100%)	25 (100%)	*–*
Cumulative corticosteroid dose (mg/kg)^f^	536.3 ± 346.1	541.8 ± 377.9	518.5 ± 217.9	0.708
Cyclophosphamide, *n* (%)	48 (45.3)	35 (43.2)	13 (52.0)	0.440
Cumulative cyclophosphamide (×10^3^ mg/m^2^)^b^	5.7 (4.2, 7.6)	5.6 (4.2, 7.6)	5.9 (3.8, 7.8)	0.721
Other medications, *n* (%)	68 (64.2)	51 (63.0)	17 (68.0)	0.646
–Azathioprine	59 (55.7)	46 (56.8)	17 (68.0)	0.318
–Mycophenolate mofetil	22 (20.8)	19 (23.5)	3 (12.0)	0.217
–Cyclosporine	4 (3.8)	4 (4.9)	0 (0)	0.571
–Methotrexate	16 (15.1)	9 (11.1)	7 (28.0)	0.055

^a^*P* < 0.05 was set as significance; ^b^Median (IQR); ^c^Disease duration: the date at diagnosis to the date of achieving FAH; ^d^Total number = 101 (77 in normal growth, 24 in growth impairment); ^e^Total number = 100 (76 in normal growth, 24 in growth impairment); ^f^Prednisolone equivalent; FAH: final adult height; BMI: body mass index; parent-adjusted final adult height *Z*-score: difference between final adult height and mid parental height *Z*-score; SLEDAI: Systemic Lupus Erythematosus Disease Activity Index; 25-OH vitamin D: 25-hydroxy-vitamin D

### Predictors of growth impairment

In the logistic regression analysis, the risk factors for growth impairment were being male and having a longer time prior to menarche (girls) and adult voice appearance (boys) (Table 2). Poor height at diagnosis and high cumulative corticosteroid doses were not significant predictors of growth impairment in the multivariate analysis. When we analyzed data from time at diagnosis to the late phase of puberty by multivariate analysis, we found that male sex (OR 8.87, 95% CI 2.12–37.17; *P* = 0.003) and cumulative dose of corticosteroids (prednisolone equivalent) of 230 mg/kg or greater (OR 6.99, 95% CI 1.63–30.02; *P* = 0.009) were predictors of growth impairment (Table 2).

**Table 2:** Predictors of growth impairment in patients with cSLE

Predictive factors	Univariate		Multivariate	
	OR (95% CI)	***P*** value	OR (95% CI)	***P*** value
**From time at diagnosis to achieving FAH (*N*** **= 106)**				
Male	5.33 (1.85–15.37)	0.002^a^	7.07 (2.11–23.74)	0.002^a^
Duration of disease prior to menarche/adult voice appearance (y)	1.27 (1.05–1.53)	0.016^a^	1.26 (1.02–1.56)	0.033^a^
Cumulative dose of corticosteroids ≥300 mg/kg^b^	3.87 (1.07–14.08)	0.040^a^	3.91 (0.93–16.37)	0.058
Height impairment at diagnosis^c^	3.00 (1.04–8.59)	0.041^a^	3.07 (0.92–10.25)	0.068
**From time at diagnosis to the late phase of puberty (*N*** **= 76)**				
Male	5.14 (1.59–16.62)	0.006^a^	8.87 (2.12–37.17)	0.003^a^
Cumulative dose of corticosteroids ≥230 mg/kg^b^	4.10 (1.22–13.75)	0.022^a^	6.99 (1.63–30.02)	0.009^a^

^a^*P* < 0.05 was set as significance; ^b^Prednisolone equivalent; ^c^Height impairment: height *Z*-score less than −1.5; FAH: final adult height

### Sex difference

**Fig. 1:**
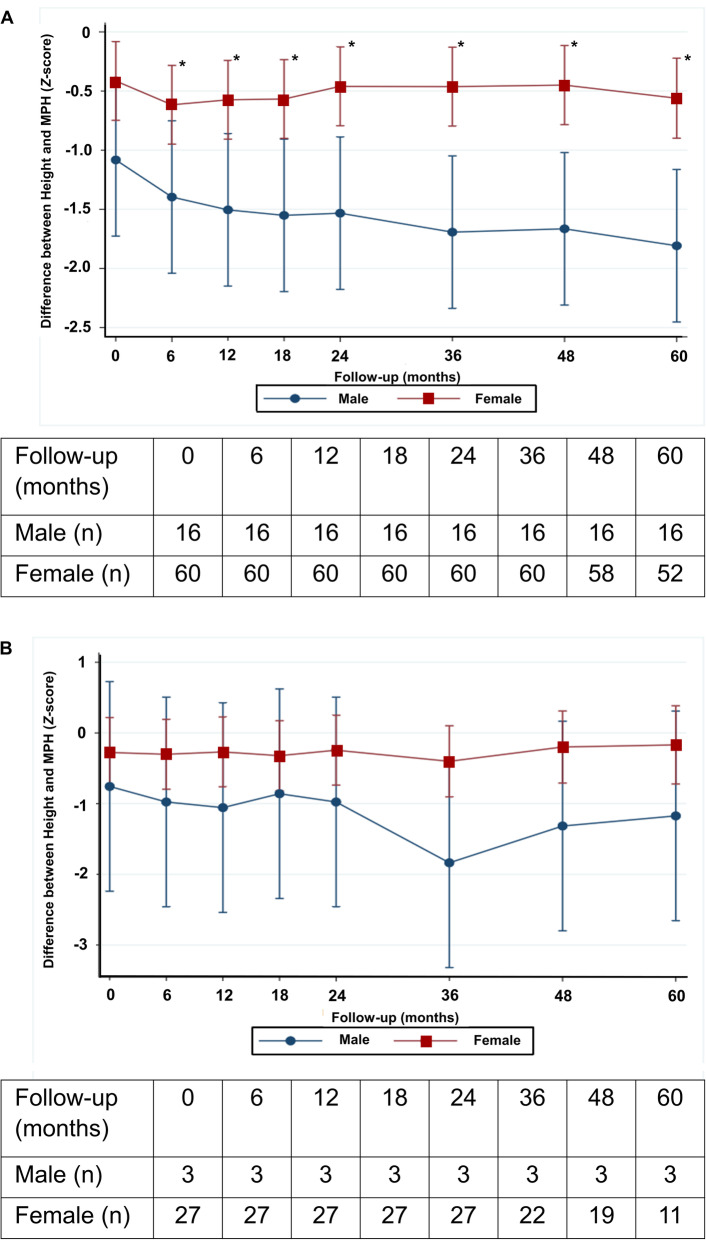
Growth trajectory pattern between male and female SLE patients. **A**) Duration between the time of the diagnosis and time of menarche in girls or adult voice appearance in boys. **B**) Duration between the time of puberty and achievement of final adult height. Data represent the mean and standard error and were analyzed by mixed-effect regression analysis. *P < 0.05

**Fig. 2:**
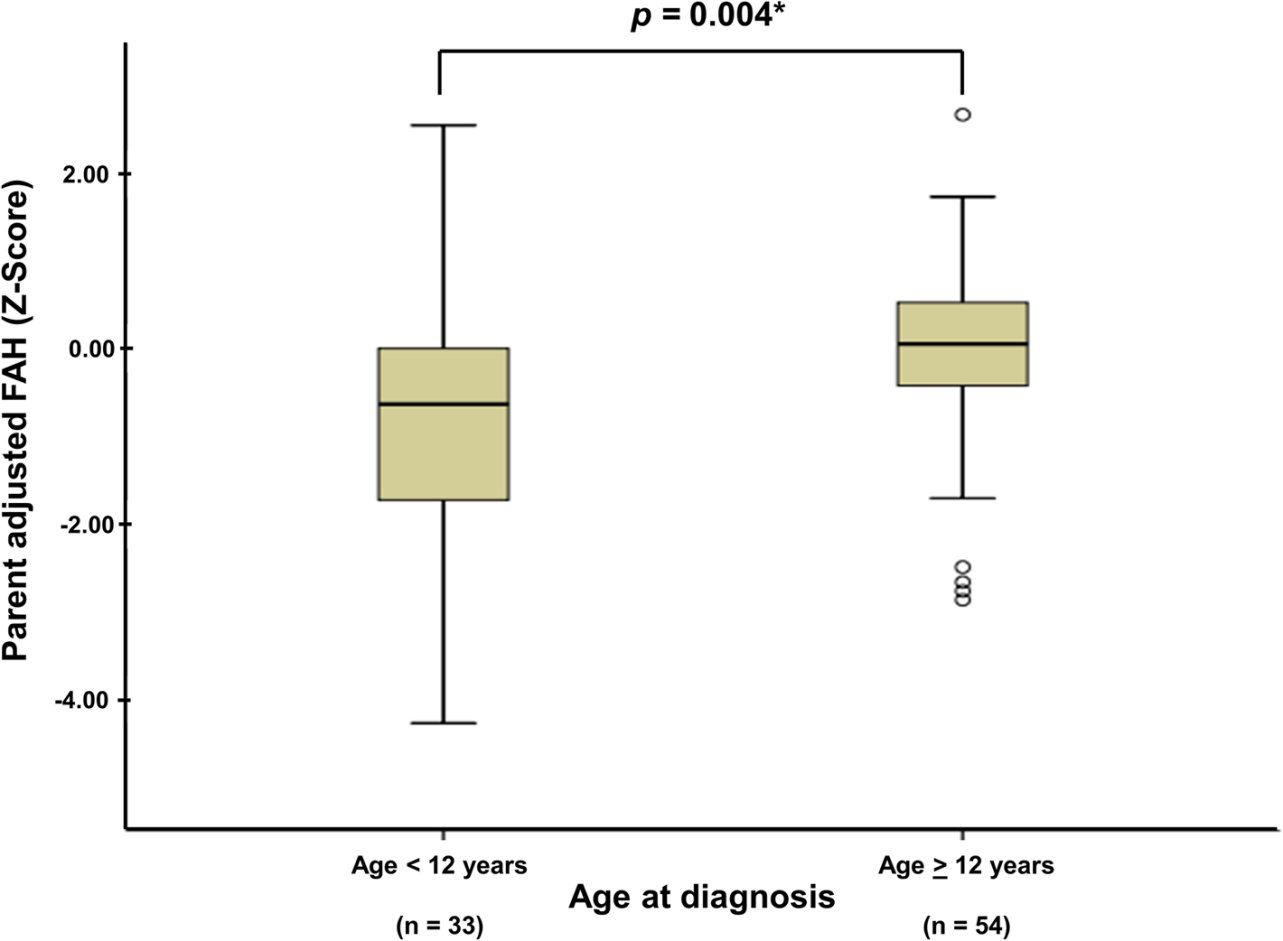
Comparison of parent-adjusted final adult height (FAH) *Z*-score between female SLE patients aged < 12 years and ≥ 12 years at diagnosis. Parent-adjusted FAH *Z*-score: difference between FAH *Z*-score for chronological age and mid-parental height *Z*-score. *P < 0.05

Age at diagnosis between boys (12.4 ± 1.7 years) and girls (12.0 ± 2.5 years) was not significant (*P* = 0.477), but age at menarche in girls (13.6 ± 1.6) and adult voice appearance in boys (14.5 ± 1.0) were both significantly different (*P* = 0.001). Approximately 52.6% of male patients had growth impairment, compared with 17.2% of female patients (*P* = 0.002). Median (IQR) PaFAH *Z*-score was significantly lower in male patients in comparison with female counterparts (− 1.7 [− 2.3, − 0.5] vs. − 0.2 [− 1.1, 0.4]; *P* < 0.001). Mean difference of height *Z*-score of male patients declined significantly more than that of female patients, especially during the period prior to the late phase of puberty. By contrast, the mean differences of height *Z*-scores were compatible between both sexes after the late phase of puberty (Fig. 1). This study also demonstrated that median (IQR) PaFAH *Z*-score was statistically lower in female patients who were diagnosed when younger than 12 years (− 0.6 [− 1.8, 0.1]) compared with counterparts diagnosed at age 12 years or older (0.1 [− 0.4, 0.5]) (*P* = 0.004) (Fig. 2). There was no specific age difference with regard to PaFAH *Z*-score in male patients. Clinical manifestations, disease duration, disease activity, and use of other medications between both sexes were not statistically different (Table 3). Predictors of growth impairment in each sex were then analyzed (Table 4). In male cSLE patients, receiving corticosteroid (prednisolone equivalent) dosing of ≥330 mg/kg was a predictor of growth impairment, while in female counterparts height impairment at diagnosis and age less than 12 years at diagnosis were predictors of growth impairment.

**Table 3:** Patient characteristics in male and female SLE patients (*N* = 106)

	Male	Female	***P*** value
	***n*** = 19	***n*** = 87	
**Patient characteristics**			
Age at diagnosis (y)	12.4 ± 1.7	12.0 ± 2.5	0.477
Age at FAH (y)	17.9 ± 1.7	17.4 ± 1.9	0.308
Age at menarche/adult voice appearance (y)	14.5 ± 1.0	13.6 ± 1.6	0.001^a^
Disease duration (y)^c^	5.7 ± 1.9	5.4 ± 2.7	0.701
Disease duration before menarche/adult voice appearance (y)^b^	2.0 (1.0, 3.5)	1.4 (0.0, 3.1)	0.314
Diagnosis before menarche, *n* (%)	–	60 (69.0)	
Diagnosis before adult voice appearance, *n* (%)	16 (84.2)	–	0.181
Cumulative Modified SLEDAI-2 K scores^b^	117.0 (66.0, 177.0)	165.0 (75.0, 267.0)	0.526
**Anthropometric data at diagnosis and at FAH attainment**			
Weight at diagnosis (*Z*-score)^b^	0.4 (−0.5, 1.6)	−0.2 (−0.9, 1.0)	0.257
Final weight (*Z*-score)^b^	0.3 (−1.4, 4.8)	0.7 (− 0.7, 3.0)	0.821
Height at the diagnosis (*Z*-score)^b^	0.0 (−0.9, 0.9)	−0.3 (−1.0, 0.7)	0.454
Final height (*Z*-score)^b^	−0.3 (−1.3, 0.3)	−0.02 (−1.3, 0.4)	0.662
BMI at diagnosis (kg/m^2^) (*Z*-score)^b^	−0.2 (−1.2, 1.6)	−0.4 (−1.5, 1.0)	0.327
Final BMI (kg/m^2^) (*Z*-score)^b^	0.1 (−1.1, 2.0)	0.4 (−0.6, 1.5)	0.618
Parent-adjusted FAH (*Z*-score)^b^	−1.7 (−2.3, −0.5)	−0.2 (−1.1, 0.4)	< 0.001^a^
Difference between FAH and MPH (cm)^b^	−9.1 (−12.2, − 2.5)	−0.8 (−5.0, 1.9)	< 0.001^a^
**Clinical manifestations at diagnosis**			
Modified SLEDAI-2 K score	19.2 ± 10.2	15.4 ± 10.7	0.156
Neurological, *n* (%)	8 (42.1)	24 (27.6)	0.212
All renal involvement, *n* (%)	14 (73.7)	54 (62.1)	0.467
Lupus nephritis class III, IV, V, *n* (%)	8 (42.1)	29 (33.3)	0.277
Musculoskeletal, *n* (%)	8 (42.1)	47 (54)	0.346
Hematological, *n* (%)	10 (52.6)	37 (42.5)	0.422
Vasculitis, *n* (%)	3 (15.8)	15 (17.2)	1.000
Serositis, *n* (%)	7 (36.8)	20 (23)	0.248
**Laboratory data at diagnosis**			
Hematocrit (%)	31.3 ± 6.6	29.6 ± 7.0	0.307
White blood cell count (×10^3^ cells/mm^3^)^b^	6.0 (3.1, 7.9)	4.7 (3.4, 6.6)	0.656
Absolute lymphocyte count (× 10^3^ cells/mm^3^)^b^	1.1 (0.7, 2.6)	1.5 (1.1, 2.1)	0.376
Platelet count (×10^3^ cells/mm^3^)^b^	2.2 (1.3, 2.8)	2.3 (1.1, 3.0)	0.934
Erythrocyte sedimentation rate (mm/h)^b^	44.0 (28.0, 57.0)	60.0 (23.0, 89.0)	0.227
Anti-dsDNA (IU/mL)^b,d^	240.7 (138.9, 609.5)	234.2 (90.6, 566.2)	0.709
C3 levels (0.9–1.8 g/L)^b^	0.4 (0.2, 1.0)	0.7 (0.4, 1.0)	0.036
C4 levels (0.1–0.4 g/L)^b^	0.07 (0.03, 1.27)	0.08 (0.05, 1.52)	0.463
25-OH Vitamin D levels (ng/mL)^e^	35.06 ± 11.41	33.52 ± 7.62	0.489
**Medications**			
Prednisolone, *n* (%)	19 (100)	87 (100)	*–*
Cumulative corticosteroid dose (mg/kg)^f^	445.8 ± 219.3	553.4 ± 367.3	0.091
Cyclophosphamide, *n* (%)	9 (47.4)	39 (44.8)	0.873
Cumulative cyclophosphamide (×10^3^ mg/m^2^)^b^	6.3 (4.7, 6.5)	5.6 (4.1, 7.8)	0.735
Other medications, *n* (%)	14 (73.7)	54 (62.1)	0.339
–Azathioprine	10 (52.6)	53 (60.9)	0.505
–Mycophenolate mofetil	4 (21.1)	18 (20.7)	1.000
–Cyclosporine	1 (5.3)	3 (3.4)	0.552
–Methotrexate	3 (15.8)	13 (14.9)	1.000

^a^*P* < 0.05 was set as significance; ^b^Median (IQR); ^c^Disease duration: the date of diagnosis to the date of achieving FAH; ^d^Total number = 101 (18 males, 83 females); ^e^Total number = 100 (17 males, 83 females); ^f^Prednisolone equivalent; FAH: final adult height; MPH: mid parental height; parent-adjusted final adult height *Z*-score: difference between final adult height and mid parental height *Z*-score; 25-OH vitamin D: 25-hydroxy-vitamin D

**Table 4:** Predictors of growth impairment between male and female cSLE patients

Predictive factors	Univariate		Multivariate	
	OR (95%CI)	***P*** value	OR (95%CI)	***P*** value
**Male patients (*N*** **= 19)**				
Age < 13 years	8.17 (1.03–64.94)	0.047^a^	7.89 (0.62–100.89)	0.112
Cumulative dose of corticosteroids ≥330 mg/kg^b^	18.0 (1.50–216.62)	0.023^a^	6.99 (1.63–30.02)	0.009^a^
**Female patients (*N*** **= 87)**				
Age < 12 years	4.26 (1.31–13.90)	0.016^a^	4.88 (1.39–17.13)	0.013^a^
Height impairment at the diagnosis^c^	4.13 (1.21–14.14)	0.024^a^	4.92 (1.30–18.72)	0.030^a^

^a^*P* < 0.05 was set as significance; ^b^Prednisolone equivalent; ^c^Height impairment at the diagnosis: height *Z*-score less than −1.5

## Discussion

This study focused on the growth trajectory and FAH, as well as predictors of growth impairment, in 106 Thai cSLE patients. The results demonstrated that approximately one-fourth of cSLE patients had growth impairment. Male patients were more likely to be affected, whereas female patients aged < 12 years and with height impairment at diagnosis were at high risk for developing growth impairment. The period from diagnosis to the late phase of puberty was the essential factor that influenced growth impairment. Receiving a cumulative corticosteroid dose of ≥230 mg/kg before the late phase of puberty was found to be an influential factor in growth impairment. Severity of disease, clinical manifestations, and use of other medications did not affect FAH or growth outcome.

The reported severity of growth impairment has differed among studies because of the varying definitions of growth impairment, age at diagnosis, and ethnicity of the study population [3, 6–8, 19–21]. In the longitudinal study conducted by the Paediatric Rheumatology International Trials Organization (PRINTO) [22] involving 331 juvenile SLE patients followed for 26 months, the percentages of growth impairment were 24.5 and 14.7% in boys and girls, respectively [3], lower than those found in the present study. The reasons for different outcomes between the PRINTO study and ours might be related to the different ethnicity of the study population: the PRINTO study was mostly performed in Caucasians, whereas our study population was Asian. Although there is no clear evidence that ethnicity affects growth impairment in cSLE patients, a previous multi-ethnic group study demonstrated that Asians were more likely to have growth impairment than other ethnic groups [8]. Other than ethnicity, the age at diagnosis was also an important factor. For instance, patients in a study from Oman [7] had a younger age at diagnosis (mean 6.4 ± 3.1 years) compared with our study (12.1 ± 2.3 years), resulting in a higher percentage of growth failure (32.0%) than reported herein (23.6%). Because normal children have pubertal growth spurt before menarche if female or first adult voice appearance if male, patients who received corticosteroids during this particular period will theoretically develop a greater degree of growth impairment than those who take corticosteroids after the late phase of puberty. Sontichai et al. [8] showed that the mean FAH in cSLE patients diagnosed after menarche was greater than in counterparts diagnosed before menarche. Our current results also support this finding that the median duration before menarche in girls and adult voice appearance in boys was significantly longer in patients with growth impairment than in those with normal growth, while this factor was also a predictor of growth impairment. Although a previous study showed that percentages of patients with growth failure increased with longer disease duration [19], our study found that the period before the late phase of puberty was more important than the total disease duration.

Regarding sexes, growth impairment differed between boys and girls. This study demonstrated that the age at diagnosis in both sexes was comparable, but the age at menarche in girls and adult voice appearance in boys were different because boys enter puberty around one year later than girls [23]. Our study showed that the median disease duration before adult voice appearance in boys was longer than the median disease duration before menarche in girls. Prolonged duration of corticosteroid exposure during this period might be one reason that leads to the higher percentage of male patients with growth impairment. Furthermore, normal boys usually have pubertal height velocity at around age 12.5–13.5 years, 1–2 years later than girls [24, 25]. However, there is a difference in timing of peak height velocity among ethnic groups, and the alteration of growth pattern might have changed over time [25, 26]. Given that around 80% of male cSLE patients in this study were diagnosed before or during the peak growth spurt (mean age at diagnosis 12.4 ± 1.7), this can explain why male patients were more affected than females and why male sex was a strongly significant predictor of growth impairment. In addition, it is not surprising that girls aged < 12 years carried a higher risk of height impairment in the present study. Because their age at menarche was approximately 13.6 years, pubertal growth spurt should have been present during age 11–13 years. Our findings regarding the difference in growth impairment between male and female cSLE patients were in line with the PRINTO study [3] but in contrast to that by Heshin-Bekenstein et al. [6], which did not find a difference in growth between sexes. This might be because the age at diagnosis of cSLE patients in that study [6] (14.0 ± 3.0 years) was older than that reported in the PRINTO study [3] and ours; therefore, their patients might already have reached FAH or were near FAH.

Currently there are limited data on the cumulative dose of corticosteroids that affects growth in cSLE patients. The PRINTO study [3] reported that a cumulative dose of 426 mg/kg of corticosteroid (prednisolone equivalent) was a predictor of growth failure with OR 3.6. The present study showed that the cumulative dose of corticosteroids for the entire disease course in both normal height and height impairment groups was not statistically different. Instead, a cumulative dose of corticosteroid of ≥230 mg/kg receiving before the late phase of puberty was a predictor of growth impairment with an OR of 6.99, whereas a cumulative dose of corticosteroids in patients diagnosed after menarche or adult voice appearance did not have a notable impact on growth development. Generally, the effect of corticosteroids on growth should depend on the cumulative dose of corticosteroid. The longer the corticosteroid use and the higher the dose, the greater the height suppression. However, this finding demonstrated that the timing of corticosteroid treatment initiation was another important factor for growth impairment, other than cumulative dose of corticosteroid. Therefore, we should be aware of cumulative corticosteroid dosing in the treatment of cSLE patients, especially when they have received corticosteroid treatment before the late phase of puberty. Furthermore, administering a higher corticosteroid dose to male patients should also be concern because according to our analysis a cumulative dose of ≥330 mg/kg also constitutes a risk factor for growth impairment in males.

There are noteworthy strengths in this study. First, our patients were longitudinally followed up from the time of the diagnosis to achievement of FAH. Second, we used the PaFAH *Z*-score, which was adjusted with MPH *Z*-score, for the outcome of growth impairment. By contrast, most previous studies did not use FAH as an outcome [3, 7, 19–21]. Since height in children depends on the genetic background of their parents and FAH was a more accurate indicator of irreversible damage than height velocity, the use of PaFAH Z-score was a more reliable tool for assessing growth outcome and reduced confounding factors. Third, we collected cumulative corticosteroid dose and assembled the sum of Modified SLEDAI-2 K scores for the entire disease duration, which is more reliable than one-time data collection. However, this study also has some limitations. First, owing to its retrospective study design, Tanner staging was not available. The period of menarche and adult voice appearance were gathered from interview data, which could be a recall bias. Second, there was no detailed nutritional history for our patients. Nevertheless, the average BMI between patients with and without growth impairment was not different. Moreover, all patients routinely received calcium and vitamin D supplements to maintain a 25-hydroxyvitamin D level of ≥30 ng/mL.

## Conclusions

One-fourth of cSLE patients developed growth impairment whereby boys were more affected than girls. The duration of corticosteroid exposure and the cumulative dose of corticosteroid administration before the late phase of puberty were important factors that affected growth development. Although control of cSLE is essential, the complications following treatment should be of concern to physicians. Decreasing corticosteroid use and choosing alternative immunosuppressive medications to control the disease may prevent further damage [27]. Given that growth is an essential indicator of children’s well-being, early recognition and prevention of growth impairment are crucial aspects of patient care [28], particularly in patients with chronic illnesses such as cSLE.

## Data Availability

The datasets analyzed during the current study are not publicly available due to a confidentiality agreement with the participants but are available from the corresponding author on reasonable request.

## Abbreviations

anti-dsDNA: Anti-double-stranded DNA
BMI: Body mass index
cSLE: Childhood-onset systemic lupus erythematosus
FAH: Final adult height
IQR: Interquartile range
OR: Odds ratio
PRINTO: Paediatric rheumatology international trials organization
SD: Standard deviation
SLE: Systemic lupus erythematosus

## References

[1] Kamphuis S, Silverman E (2010). Prevalence and burden of pediatric-onset systemic lupus erythematosus. Nat Rev Rheumatol.

[2] Hiraki L, Hamilton J, Silverman E (2007). Measuring permanent damage in pediatric systemic lupus erythematosus. Lupus.

[3] Rygg M, Pistorio A, Ravelli A, Maghnie M, Di Iorgi N, Bader-Meunier B (2012). A longitudinal PRINTO study on growth and puberty in juvenile systemic lupus erythematosus. Ann Rheum Dis.

[4] Mushtaq T, Ahmed S (2002). The impact of corticosteroids on growth and bone health. Arch Dis Child.

[5] Shiff N, Brant R, Guzman J, Cabral DA, Huber AM, Miettunen P (2013). Glucocorticoid-related changes in body mass index among children and adolescent with rheumatic diseases. Arthritis Care Res.

[6] Heshin-Bekenstein M, Perl L, Hersh AO, von Scheven E, Yelin E, Trupin L, Yazdany J, Lawson EF (2018). Final adult height of patients with childhood-onset systemic lupus erythematosus: a cross sectional analysis. Pediatr Rheumatol Online J..

[7] Abdalla E, Jeyaseelan L, Ullah I, Abdwani R (2017). Growth pattern in children with systemic lupus erythematosus. Oman Med J.

[8] Sontichai W, Liao F, Dominguez D, Levy DM, Al Mutairi M, Ng L, et al. Timing of childhood-onset systemic lupus erythematosus diagnosis relative to menarche impacts final height. Arthritis Care Res (Hoboken). 2020. 10.1002/acr.24461. 10.1002/acr.24461 32976694

[9] Hiraki LT, Benseler SM, Tyrrell PN, Harvey E, Hebert D, Silverman ED (2009). Ethnic differences in pediatric systemic lupus erythematosus. J Rheumatol.

[10] Hochberg MC (1997). Updating the American College of Rheumatology revised criteria for the classification of systemic lupus erythematosus. Arthritis Rheum.

[11] Petri M, Orbai AM, Alarcón GS, Gordon C, Merrill JT, Fortin PR, Bruce IN, Isenberg D, Wallace DJ, Nived O, Sturfelt G, Ramsey-Goldman R, Bae SC, Hanly JG, Sánchez-Guerrero J, Clarke A, Aranow C, Manzi S, Urowitz M, Gladman D, Kalunian K, Costner M, Werth VP, Zoma A, Bernatsky S, Ruiz-Irastorza G, Khamashta MA, Jacobsen S, Buyon JP, Maddison P, Dooley MA, van Vollenhoven RF, Ginzler E, Stoll T, Peschken C, Jorizzo JL, Callen JP, Lim SS, Fessler BJ, Inanc M, Kamen DL, Rahman A, Steinsson K, Franks AG, Sigler L, Hameed S, Fang H, Pham N, Brey R, Weisman MH, McGwin G, Magder LS (2012). Derivation and validation of the systemic lupus international collaborating clinics classification criteria for systemic lupus erythematosus. Arthritis Rheum.

[12] Harries MLL, Walker JM, Williams DM, Hawkins S, Hughes IA (1997). Changes in the male voice at puberty. Arch Dis Child.

[13] Greulich WW, Pyle SI (1959). Radiographic atlas of skeletal development of the hand and wrist.

[14] Uribe AG, Vilá LM, McGwin G, Sanchez ML, Reveille JD, Alarcón GS (2004). The systemic lupus activity measure—revised, the Mexican systemic lupus erythematosus disease activity index (SLEDAI), and a modified SLEDAI-2K are adequate instruments to measure disease activity in systemic lupus erythematosus. J Rheumatol.

[15] Brunner HI, Silverman ED, Bombardier C, Feldman BM (2003). European consensus lupus activity measurement is sensitive to change in disease activity in childhood-onset systemic lupus erythematosus. Arthritis Rheum.

[16] de Onis M, Onyango AW, Borghi E, Siyam A, Nishida C, Siekmann J (2007). Development of a WHO growth reference for school-aged children and adolescents. Bull World Health Organ.

[17] Department of Health, Ministry of Public Health (Thailand) (2000). Working group on using weight and height references in evaluating the growth status of Thai children. Manual on using weight and height references in evaluating the growth status of Thai children. Bangkok.

[18] Growth Hormone Research Society (2000). Consensus guidelines for the diagnosis and treatment of growth hormone (GH) deficiency in childhood and adolescence: summary statement of the GH research society. J Clin Endocrinol Metab.

[19] Gutiérrez-Suárez R, Ruperto N, Gastaldi R, Pistorio A, Felici E, Burgos-Vargas R, Martini A, Ravelli A, Pediatric Rheumatology International Trials Organization (PRINTO) (2006). A proposal for a pediatric version of the systemic lupus international collaborating clinics/American College of Rheumatology Damage Index based on the analysis of 1,015 patients with juvenile-onset systemic lupus erythematosus. Arthritis Rheum.

[20] Sit JKK, Chan WKY (2018). Risk factors for damage in childhood-onset systemic lupus erythematosus in Asians: a case control study. Pediatr Rheumatol Online J.

[21] Bandeira M, Buratti S, Bartoli M, Gasparini C, Breda L, Pistorio A, Grassi S, Alpigiani MG, Barbano G, Janz-Junior LL, Martini A, Ravelli A (2006). Relationship between damage accrual, disease flares and cumulative drug therapies in juvenile-onset systemic lupus erythematosus. Lupus..

[22] Ruperto N, Martini A (2004). International research networks in pediatric rheumatology: the PRINTO perspective. Curr Opin Rheumatol.

[23] Granados A, Gebremariam A, Lee JM (2015). Relationship between timing of peak height velocity and pubertal staging in boys and girls. J Clin Res Pediatr Endocrinol.

[24] Marshall WA, Tanner JM (1970). Variations in the pattern of pubertal changes in boys. Arch Dis Child.

[25] Zheng W, Suzuki K, Yokomichi H, Sato M, Yamagata Z (2013). Multilevel longitudinal analysis of sex differences in height gain and growth rate changes in Japanese school-aged children. J Epidemiol.

[26] Backelijauw PF, Dattani MT, Cohen P, Rosenfeld RG, Sperling MA (2014). Disorders of growth hormone/insulin-like growth factor secretion and action. Pediatric endocrinology.

[27] Brunner HI, Silverman ED, Bombardier C, Feldman BM, To T (2002). Risk factors for damage in childhood-onset systemic lupus erythematosus: cumulative disease activity and medication use predict disease damage. Arthritis Rheum.

[28] Zeitler PS, Travers S, Kappy MS (1999). Advances in the recognition and treatment of endocrine complications in children with chronic illness. Adv Pediatr Infect Dis.

